# Thrombophlebitis of the Right Renal Capsular Vein during the Early Postpartum Period

**DOI:** 10.1155/2018/3096468

**Published:** 2018-10-09

**Authors:** Koji Nakamura, Kensuke Nakanishi, Satoshi Kubota, Ryoko Takahashi, Mari Tomiie, Akihiro Moriyama

**Affiliations:** ^1^Department of Obstetrics and Gynecology, Osaka Saiseikai Nakatsu Hospital, 2-10-39 Shibata, Kita-ku, Osaka 530-0012, Japan; ^2^Department of Molecular Oncology, H. Lee Moffitt Cancer Center and Research Institute, Tampa, FL, USA; ^3^Department of General and Gastroenterological Surgery, Osaka Medical College, Takatsuki, Osaka, Japan; ^4^Department of Obstetrics and Gynecology, Toyonaka Municipal Hospital, Toyonaka, Osaka, Japan

## Abstract

Venous thrombophlebitis is an uncommon cause of fever and lower abdominal pain during the early postpartum period. It mostly occurs in the right ovarian vein, and computed tomography (CT) is useful for diagnosis. We present a case of thrombophlebitis of the renal capsular vein. A 27-year-old postpartum woman presented with right lower abdominal pain and fever unresponsive to antibiotics. Contrast CT showed a ring-enhancing mass in the right retroperitoneum, which was distinct from the right ovarian vein. Exploratory laparoscopy revealed a retroperitoneal hematoma and normal appendix. Reconstruction of CT images revealed that the mass was connected to the right renal capsular vein. Anticoagulation therapy improved the patient's symptoms. Postpartum thrombophlebitis can occur at locations other than the ovarian vein, such as the renal capsular vein. If a retroperitoneal mass is discovered during puerperium, a thorough investigation of the mass's continuity with surrounding vessels is essential to avoid unnecessary surgery.

## 1. Introduction

Venous thrombophlebitis is an uncommon cause of fever and lower abdominal pain during the early postpartum period [1]. Almost all previously reported cases have occurred in the ovarian vein, especially the right ovarian vein [2]. Imaging studies such as computed tomography (CT) and magnetic resonance imaging (MRI) are useful for diagnosis [3]. In this report, we present an extremely rare case of thrombophlebitis of the right renal capsular vein occurring during the early postpartum period.

## 2. Case Presentation

**Figure 1:**
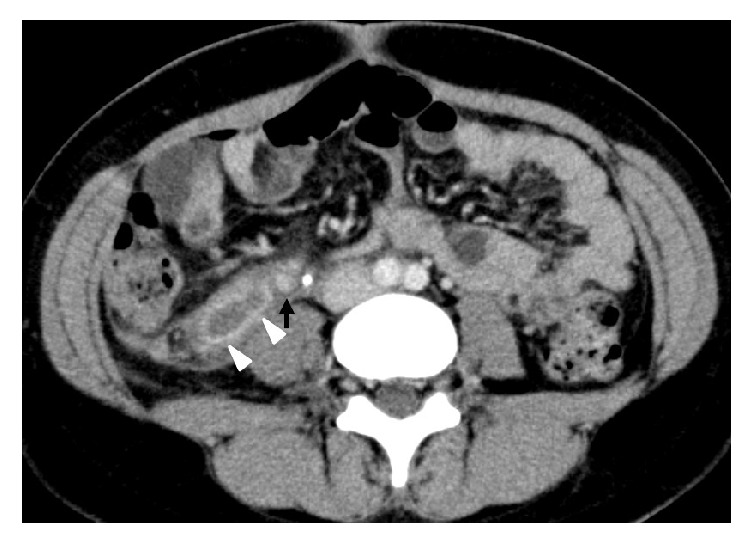
Abdominal dynamic CT scan (delayed phase). A ring-enhanced right retroperitoneal mass (white arrowhead). It was distinct from the right ovarian vein (black arrow).

**Figure 2:**
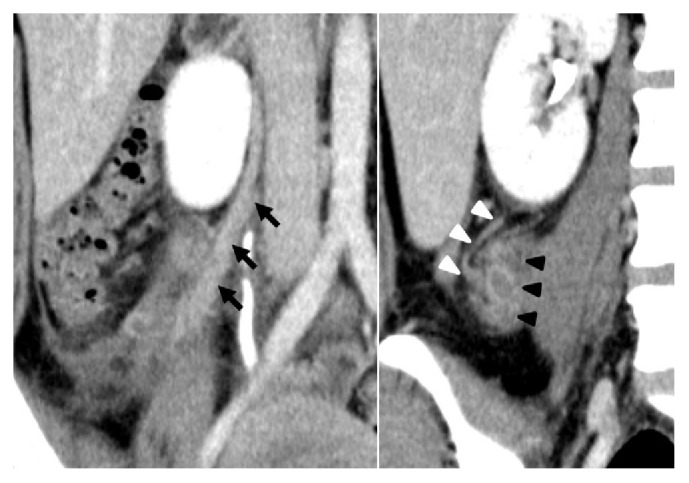
Abdominal dynamic CT scan reconstructed after surgery (delayed phase). Intact right ovarian vein (black arrow) (left). The retroperitoneal mass (black arrowhead) was connected to the right renal capsular vein (white arrowhead) (right).

**Figure 3:**
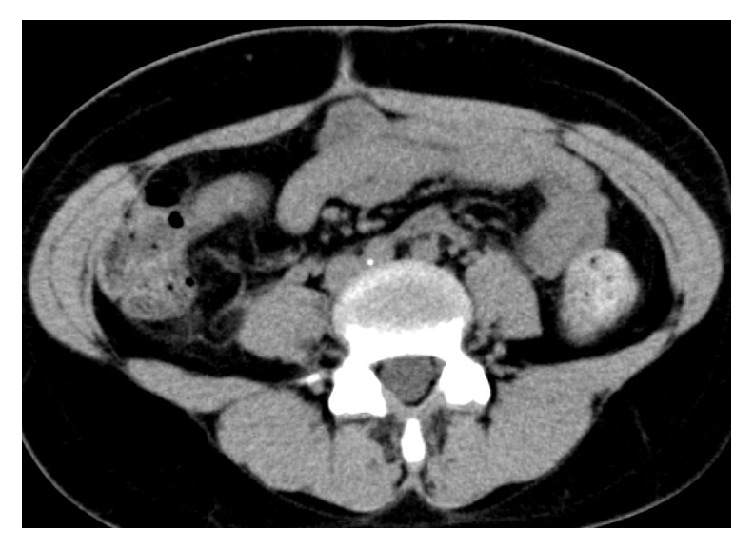
Abdominal plain CT scan one month after surgery. The retroperitoneal mass had disappeared.

A healthy 27-year old woman without any history of thrombosis or thrombophlebitis, gravida 1 para 0, conceived spontaneously. There were no problems with the course of her pregnancy. At 40 weeks of gestation, she vaginally delivered a healthy 3156-g baby, and vacuum extraction was needed because of a nonreassuring fetal status. On the fourth day after delivery, she complained of a 38.5 degree fever and lower abdominal pain. The right side of her uterine fundus was tender to palpation. There was no rebound pain or muscular defense. There was no evidence of infection in her perineal laceration. Transabdominal ultrasonography showed no abnormal findings. Her blood profile was as follows: white blood cells, 12600/mm^3^ (normal range: 4000-9000/mm^3^); C-reactive protein, 2.2 mg/L (normal range: <0.3mg/L). Her urine profile was unremarkable. She was treated with antibiotics (1.5 g ampicillin/sulbactam 4 times per day) according to a diagnosis of postpartum endometritis. Her symptoms persisted despite the antibiotic treatment. On the seventh day after delivery, her right lower abdominal pain worsened, and rebound pain appeared as well. In addition, right costovertebral angle tenderness appeared. There was no tenderness in her uterine fundus. Her blood and urine profiles showed no remarkable changes. On the eighth day, an abdominal dynamic CT scan showed a sausage-like, ring-enhanced right retroperitoneal mass with a diameter of 27 mm, which was distinct from the right ovarian vein (Figure 1). Retroperitoneal appendicitis was suspected, and exploratory laparoscopy was performed on the ninth day. During the surgery, a normal appendix and right ovarian vein were identified. The peritoneum located on the dorsal side of the appendix was bulging. When the peritoneum was incised, blood clots appeared on the right side of the right ovarian vein. No additional pathologic processes were identified. After the surgery, we reconstructed the CT images for further investigation. The coronal reconstructed CT images revealed that the retroperitoneal mass was connected to the right renal capsule vein (Figure 2). Based on a diagnosis of thrombophlebitis of the right renal capsular vein, anticoagulant treatment (2000 IU subcutaneous enoxaparin twice per day) was started on the tenth day. Antibiotics were stopped because no signs of infection were observed during the surgery. Her temperature returned to normal on the fourth day of anticoagulation, and her abdominal pain disappeared on the sixth day. On the seventh day, anticoagulation was stopped, and she was discharged from our hospital. After a month of follow-up, she was still in good condition, and the CT showed that the mass had disappeared (Figure 3).

## 3. Discussion

In this case, we made two important clinical observations. First, postpartum thrombophlebitis can occur in the renal capsule vein. Second, CT scanning can be useful for the diagnosis of this disease.

Postpartum thrombophlebitis can occur in the renal capsule vein. Such pelvic or abdominal venous thrombophlebitis is a rare but serious postpartum complication. This disease is characterized by inflammation or thrombosis of a pelvic or abdominal vein, and patients typically present with fever and abdominal pain within the first postpartum week [4]. Almost all previous reports have been regarding ovarian vein thrombophlebitis. The incidence of this pathology is reported to range from 1/600 to 1/2000 pregnancies [1], and it occurs in the right ovarian vein in 90% of cases [2]. Several factors are considered to be responsible for this distribution. First, compression of the right ovarian vein due to the dextroversion of the enlarged uterus may lead to venous stasis [2]. Second, the right ovarian vein is longer than the left and has several valves, which may act as niduses for thrombus formation [2]. In contrast, there have been very few case reports of patients with postpartum venous thrombophlebitis in veins other than the ovarian vein, such as the mesenteric or portal veins [5, 6]. In this report, we present a case of thrombophlebitis occurring in the right renal capsular vein. To the best of our knowledge, this is the first report of this status. The renal capsular vein is a minor variation of normal renal vasculature. It is defined as a tributary of the renal vein that drains blood from the renal capsule. The renal capsular veins may have extra- or intrarenal communication; however, these veins typically flow into the adrenal vein [7]. Thrombophlebitis is associated with Virchow's triad of venous stasis, hypercoagulability, and vessel wall injury. In fact, all three components of Virchow's triad occur in the course of pregnancy and delivery [8]. Although the exact cause of involvement of the right renal capsular vein in this case remains unclear, it is possible that the venous endothelium was accidentally damaged by the instrumental delivery.

CT scanning can be useful for the diagnosis of postpartum thrombophlebitis of the renal capsular vein. Since symptoms of this disease are nonspecific, such as fever and lower abdominal pain, patients tend to be initially diagnosed with postpartum endometritis and administered antibiotics. If the symptoms do not improve after initial antibiotic treatment, further evaluation is indicated. The differential diagnosis includes venous thrombophlebitis, infected hematoma, pelvic abscess, surgical site infection, and appendicitis, which may occur during puerperium [9]. Imaging studies such as CT and MRI have been reported to be useful for the diagnosis of ovarian vein thrombophlebitis. According to a previously published case series, their sensitivities are 100% and 92%, and their specificities are 99% and 100%, respectively [3]. In this case, however, appendicitis existing in the retroperitoneum or spreading to the retroperitoneum was initially suspected rather than venous thrombophlebitis. This was based on several specific imaging findings. First, the right retroperitoneal mass was distinct from the right ovarian vein. Second, the appendix was not identified in the peritoneal cavity. Third, the mass looked like a luminal structure. Although appendicitis in the early postpartum period is also difficult to diagnose [10, 11], in this case, the surgical findings were confirmed by reconstruction of CT images. If this reconstruction had been performed before the surgery, exploratory laparoscopy could have been avoided. Therefore, contrast CT scanning can be considered to be a vital tool for the diagnosis of thrombophlebitis of the renal capsular vein. As a lesson from this case, we have considered other approaches to distinguish venous thrombophlebitis from appendicitis. A meta-analysis showed that MRI has the potential to be the first-line diagnostic test due to its high accuracy (sensitivity and specificity are both 96%) [12]. A case report showed that CT angiography can be a diagnostic option by enabling direct visualization of the thrombus [13]. Therefore, a combination of these diagnostic tests may improve diagnostic accuracy.

In conclusion, postpartum thrombophlebitis can occur in the renal capsular vein. Although this condition is extremely rare, it should be considered when evaluating postpartum patients who display persistent fever and lower abdominal pain. Imaging modalities, including CT scanning, can be useful in the diagnosis of this disease. However, meticulous evaluation is essential in order to avoid misdiagnosis and unnecessary surgery.
